# Research progress on the role of inflammatory mediators in the pathogenesis of epilepsy

**DOI:** 10.1002/ibra.12162

**Published:** 2024-05-29

**Authors:** Yue Yu, Fei‐Ji Sun

**Affiliations:** ^1^ Department of Neurosurgery Affiliated Hospital of Zunyi Medical University Zunyi China; ^2^ Department of Neurosurgery The First Affiliated Hospital of Chongqing Medical and pharmaceutical college Chongqing China

**Keywords:** epilepsy, immune reaction, inflammatory mediators, inflammatory signal pathway, seizure

## Abstract

Epilepsy is an abnormal neurologic disorder distinguished by the recurrent manifestation of seizures, and the precise underlying mechanisms for its development and progression remain uncertain. In recent years, the hypothesis that inflammatory mediators and corresponding pathways contribute to seizures has been supported by experimental results. The potential involvement of neuroinflammation in the development of epilepsy has garnered growing interest. This review centers attention on the involvement of inflammatory mediators in the emergence and progression of epilepsy within recent years, focusing on both clinical research and animal models, to enhance comprehension of the intricate interplay between brain inflammation and epileptogenesis.

## Graphical abstract

In the central nervous system, activated immune cells lead to the overproduction of inflammatory mediators through the corresponding signal pathway. Under the stimulation of inflammatory factors, neuroinflammation ultimately occurs. Overexpression of inflammatory mediators and activated immunocytes plays an important role in the emergence and progression of epilepsy. Epileptic seizure also promotes neuroinflammatory status, which may be related to drug‐resistant epilepsy.

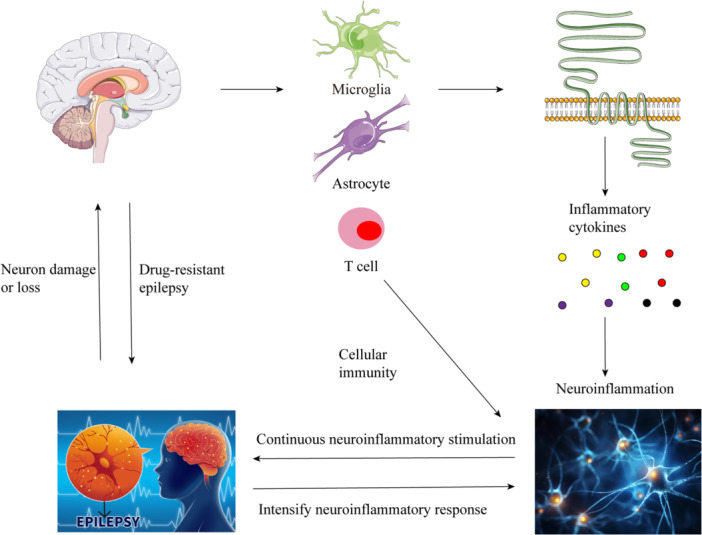

## 1 INTRODUCTION

Epilepsy is seen as a neurological disorder characterized by an underlying tendency to seizures as well as neurobiologic, cognitive, psychologic, and social consequences. Ten percent of people worldwide suffer from seizures, and 1%–2% of those individuals develop epilepsy because of seizures. The occurrence of epilepsy is related to various factors, such as traumatic brain injury, autoimmunity, illness, infections of the central nervous system, tumors, and malformations of cortical development. About 30%–40% of individuals diagnosed with epilepsy exhibit resistance to antiepileptic medications and other therapeutic interventions, resulting in ineffective seizure control.^1^ In recent years, according to a large amount of clinical and basic research, there is a close relationship between brain inflammation and the pathogenesis of epilepsy. Therefore, gaining a comprehensive understanding of the mechanism underlying inflammation holds significant importance in clarifying the onset of epilepsy.

## 2 THE RELATIONSHIP BETWEEN CENTRAL NERVOUS SYSTEM INFLAMMATION AND EPILEPSY

The blood–brain barrier (BBB) is the main regulatory structure for the interaction between the whole brain tissue cells and peripheral immune cells. Research indicates the occurrence of pericytosis during seizures and introduces a pericyte‐microglial mediated mechanism of BBB dysfunction in epilepsy.^2^ Notably, in contrast to illnesses characterized by cell surface antigens, disorders with intracellular antigens exhibit a heightened susceptibility to epilepsy.^3^ Furthermore, the damaged neurons lead to gliosis, and epilepsy is closely related to the persistent and robust inflammatory reaction in the microenvironment of nervous tissue.^4^ Clinical experiments using positron emission tomography reveal a strong correlation between seizure and acute neuroinflammation in temporal, frontal, and localized cortical dysplasia. Episodes characterized by acute neuroinflammation have the potential to induce a persistent state of neuroinflammation, hence intensifying pre‐existing chronic neuroinflammatory conditions.^5^ Under the continuous stimulation of repeated seizures or inflammatory factors induced by chronic inflammation, epilepsy may eventually occur. The phenomenon of medication resistance and the evolution of epilepsy are facilitated by the occurrence of apoptosis in glial cells and neurons.^6^

## 3 INFLAMMATORY MEDIATORS AND THE MECHANISM OF EPILEPSY OCCURRENCE

The balance of the brain's immune system is maintained in large part by cytokines. Various cells have the capability to secrete distinct cytokines, which in turn exhibit diverse biological consequences. Correlations in mediator levels within and between brain regions indicate that there are local and global regulations,^7^ which can induce epilepsy through various molecular mechanisms.

### 3.1 High‑mobility group box‐1

The subgroup analysis conducted on specimen types indicates that people diagnosed with epilepsy have increased levels of plasma high‑mobility group box‐1 (HMGB1) and cerebral spinal fluid HMGB1 in comparison to the control group.^8^ It is worth mentioning that the levels of HMGB1 in the group experiencing severe seizures exhibit an elevation when compared to both the control group and the group experiencing less severe seizures.^9^ The levels of HMGB1 are found to be higher in children who experience febrile seizures and subsequently develop epilepsy, compared to those who do not develop epilepsy.^10^ Therefore, the detection of HMGB1 in cerebrospinal fluid can predict the cause and prognosis of epilepsy,^11^ whose prognostic value is confirmed since the examination of the receiver operating characteristic curve indicates that HMGB1 has a higher level of accuracy in predicting seizure frequency compared to interleukin‐1β (IL‐1β),^12^ indicating that HMGB1 may be a critical factor in the seizure mechanism. It is reported that the release and expression of inflammatory cytokines can be downregulated by anti‐HMGB1.^13^ The positive benefits of inhibiting the HMGB‐1‐mediated signaling pathway have been shown in the context of lowering neuroinflammation and neurodegeneration following an episode of status epilepticus (SE).^14^ Notably, anti‐inflammatory treatment can improve the clinical symptoms of epilepsy.^15^ Recent research studies show that the translocation of HMGB1 in the thalamic reticular nucleus is promoted by kainic acid and inhibited by perampanel,^16^ suggesting that anti‐inflammatory treatment may become an important measure for epilepsy treatment. During the refractory SE period, HMGB1 is upregulated and translocated rapidly.^17^ However, there is less research about HMGB1 in the study of intractable epilepsy. Further exploration of its role in drug‐resistant epilepsy is of great significance.

### 3.2 Interleukin‐related inflammatory mediators

In physiological conditions, proinflammatory cytokines IL‐1β, IL‐2, and IL‐6 are expressed in small amounts in the brain.^18^ The involvement of immunocytes and associated cytokines in the pathogenesis and progression of epileptic lesions is significant.^19^ Clinical studies have found that the expression levels of IL‐1β,^20^ IL‐6,^21^ IL‐18,^20^, ^22^ and IL‐33 ^23^ in the serum of epilepsy patients are elevated. Specifically, the plasma level of IL‐1β is notably higher in patients with febrile seizures.^24^ Similarly, the levels of IL‐1β in the peripheral bloodstreams of children diagnosed with intractable temporal lobe epilepsy (TLE) exhibit a statistically significant increase in comparison to the control group.^25^ What is more, the presence of IL‐1 gene cluster variations in IL‐1β‐31 and IL‐1β‐511 has been identified as a host genetic factor that contributes to the onset of febrile seizures,^26^ since IL‐1β significantly reduces human hippocampal neurogenesis.^27^ The study findings indicate a noteworthy association between serum IL‐1β levels and medication resistance in pediatric patients diagnosed with epilepsy.^28^ Multivariate analysis shows that IL‐1β expression level is independently associated with seizure recurrence.^29^ A study from clinical specimens shows that there is a positive correlation between the presence of IL‐1β in CD14^+^ monocytes and the frequency of seizures. The production of interferon‐gamma (IFN‐γ) in Natural killer T cell (NKT)‐like cells exhibits a negative correlation with the duration of epilepsy.^30^ The above studies strongly suggest that sustained expression of IL‐1β may lead to the occurrence of drug‐resistant epilepsy.

In addition, increased circulatory concentrations of IL‐6 are associated with high glutamic acid decarboxylase antibodies (GADA) titers in patients with epilepsy, further elucidating immune mechanisms in GADA‐associated autoimmune epilepsy.^31^ IL‐6 is significantly associated with the occurrence of febrile seizures.^32^ The cerebrospinal fluid (CSF) levels of IL‐6, IL‐17, CXC chemokine ligand (CXCL) 12, and HMGB1 are significantly higher in the suspected autoimmune epilepsy (sAE) group.^33^ The antibody treatment of the IL‐6 receptor reduces seizure development and frequency in mice lacking the synapsin 2 gene when seizure before,^34^ which is the first study to investigate the effects of a systemic IL‐6 receptor antibody treatment on epilepsy development.

On the other hand, IL‐4 suppresses traumatic brain injury‐induced acceleration of epileptogenesis in rats by steering neuroinflammation toward an anti‐inflammatory state and inhibition of cell death.^35^ The administration of IL‐4 has been found to have a substantial impact on the expression of necrosis factor alpha (TNF‐α) and IL‐10 in the brain, leading to a considerable reduction in TNF‐α expression and an enhancement of IL‐10 levels.^36^ Patients with drug‐resistant epilepsy exhibit notably lower average plasma levels of IL‐10, while average serum levels of interferon‐gamma (IFN‐γ) are significantly higher.^37^ There is an observed correlation between persistently low levels of IL‐10 in the bloodstream and the presence of hippocampal sclerosis in individuals with refractory TLE. Hence, the assessment of plasma IL‐10 could potentially serve as a diagnostic biomarker for distinguishing individuals with TLE and hippocampal sclerosis from those with other forms of epilepsy.^38^ IL‐4 and IL‐10 demonstrate anti‐inflammatory properties inside the central nervous system, thereby inhibiting the development of epilepsy.

Research has shown that the deletion of IL‐17A has been observed to alleviate anxious behavior associated with TLE, possibly the safeguarding of hippocampus neurons and the reduction in aberrant neurogenesis induced by seizures.^39^ Individuals with neuropsychiatric systemic lupus erythematosus (NPSLE) exhibit increased levels of free IL‐18, particularly among those who manifest seizures in association with NPSLE,^40^ which indicates a close relationship between epilepsy and high levels of interleukin expression. Recent literature suggest that rats subjected to pentylenetetrazol (PTZ) treatment exhibit heightened seizure intensity, memory impairment, and elevated levels of TNF‐α, IL‐1β, and oxidative markers.^41^ The expression of IL‐13, RANTES,^42^ IFN‐γ, and the levels of IL‐1β ^43^ have been observed to exhibit an upward trend in the bloodstream of individuals diagnosed with autoimmune epilepsy. IFN‐ γ and IL‐1β may become important markers for diagnosing drug‐resistant epilepsy in children. Detecting the concentration of IFN‐γ and IL‐1β in serum may be related to the prognosis of pediatric epilepsy patients ^43^ (Table 1).

**Table 1:** The presence of interleukin‐related cytokine activation in clinical studies of epilepsy patients.

Cytokines	Patient population	Findings	Ref.
IL‐1β	Epilepsy surgery, 21 MTLE‐HS patients	Upregulation of IL‐1β in MTLE‐HS patients.	[20]
	18 children with RE	IL‐1β plays roles in pathophysiology in RE patients.	[43]
IL‐6	15 pediatric patients with DRE	The level of IL‐1β CD14^+^ monocytes correlated with seizure frequency.	[30]
	35 patients with PTE	IL‐6 levels were significantly higher in the PTE group.	[21]
	247 patients with epilepsy	IL‐6 concentrations were significantly higher in patients with high GADA positivity.	[31]
	Twenty patients aged ≥50 years, ten patients were diagnosed with sAE	The CSF levels of IL‐6 were significantly higher in the sAE group.	[33]
IL‐13	25 participants underwent testing. 8 were antibody‐positive	Significant elevations in the mean concentration of IL‐13 in CSF were found in the antibody positive cases.	[42]
IL‐17	20 patients aged ≥50 years, 10 patients were diagnosed with sAE	The CSF levels of IL‐17 were significantly higher in the sAE group.	[33]
IL‐18	Epilepsy surgery, 21 MTLE‐HS patients	Upregulation of IL‐18 in MTLE‐HS patients.	[20]
	119 epilepsy patients	Epilepsy patients had significantly higher serum levels of IL‐18.	[22]

Abbreviations: CSF, cerebrospinal fluid; DRE, drug‐resistant epilepsy; GADA, glutamic acid decarboxylase; IL, interleukin; MTLE‐HS, mesial temporal lobe epilepsy with hippocampal sclerosis; PTE, Posttraumatic epilepsy; RE, refractory epilepsy; sAE, suspected autoimmune epilepsy.

Basic research also finds that IL‐1β and TNF‐α have a higher expression level in drug‐resistant epileptic rats.^44^ The heightened expression of IL‐1β may potentially contribute to the development of epileptogenesis.^45^ Topiramate and thalidomide have been found to potentially prolong the time interval until the occurrence of the initial spontaneous recurring seizures (SRS), decrease SRS frequency, and decrease TNF‐α and IL‐1β concentrations in the hippocampus.^46^ Accordingly, upregulation of messenger ribonucleic acid (RNA) expression about cytokine IL‐1β, IL‐6, TNF‐α, and transforming growth factor‐β1 (TGF‐β1) has been found in the hippocampus after seizure.^18^ It suggests a high transcriptional expression of inflammatory genes during seizures.

At present, lots of studies aim to regulate interleukin‐related cytokine to relieve epilepsy. Vagus nerve stimulation has the ability to decrease the expression of IL‐1β and IL‐6. The antiepileptic mechanism of vagus nerve stimulation may be achieved by inhibiting the expression of inflammatory mediators in epileptic foci.^47^ It suggests that in addition to the conventional use of antiepileptic drugs, the combination of acupuncture, moxibustion, and electrical stimulation may achieve better results in treating epilepsy patients. In the kainic acid‐induced TLE model, G protein‐coupled receptor (GPR)120 increases in both the hippocampus and temporal lobe cortex, while the overexpression of GPR120 has been found to have a considerable mitigating effect on epileptic activity, leading to a reduction in neuronal mortality following SE. Additionally, it decreases the levels of IL‐1 β, IL‐6, and IL‐18. Conversely, the silencing of GPR120 exhibits an opposite effect.^48^ According to research findings, the introduction of miR‐10a mimics resulted in an increase in the expression levels of TNF‐α, IL‐1β, and IL‐6.^49^ Similarly, after overexpression of miR‐136 in the hippocampus tissue of epileptic rats, the level of IL‐1β in hippocampal tissue, IL‐6, and TNF‐α has significantly decreased, which can significantly reduce the number of seizures and the duration of seizures. The potential of miR‐136 to mitigate inflammation in the hippocampus of rats with epilepsy and its ability to hinder neuronal death has been observed.^50^ This study only focuses on temple lobe epilepsy, and it is worth noting whether miR‐136 plays a neuroprotective role in other types of epilepsy.

### 3.3 Chemokine‐related inflammatory mediator

**Figure 1:**
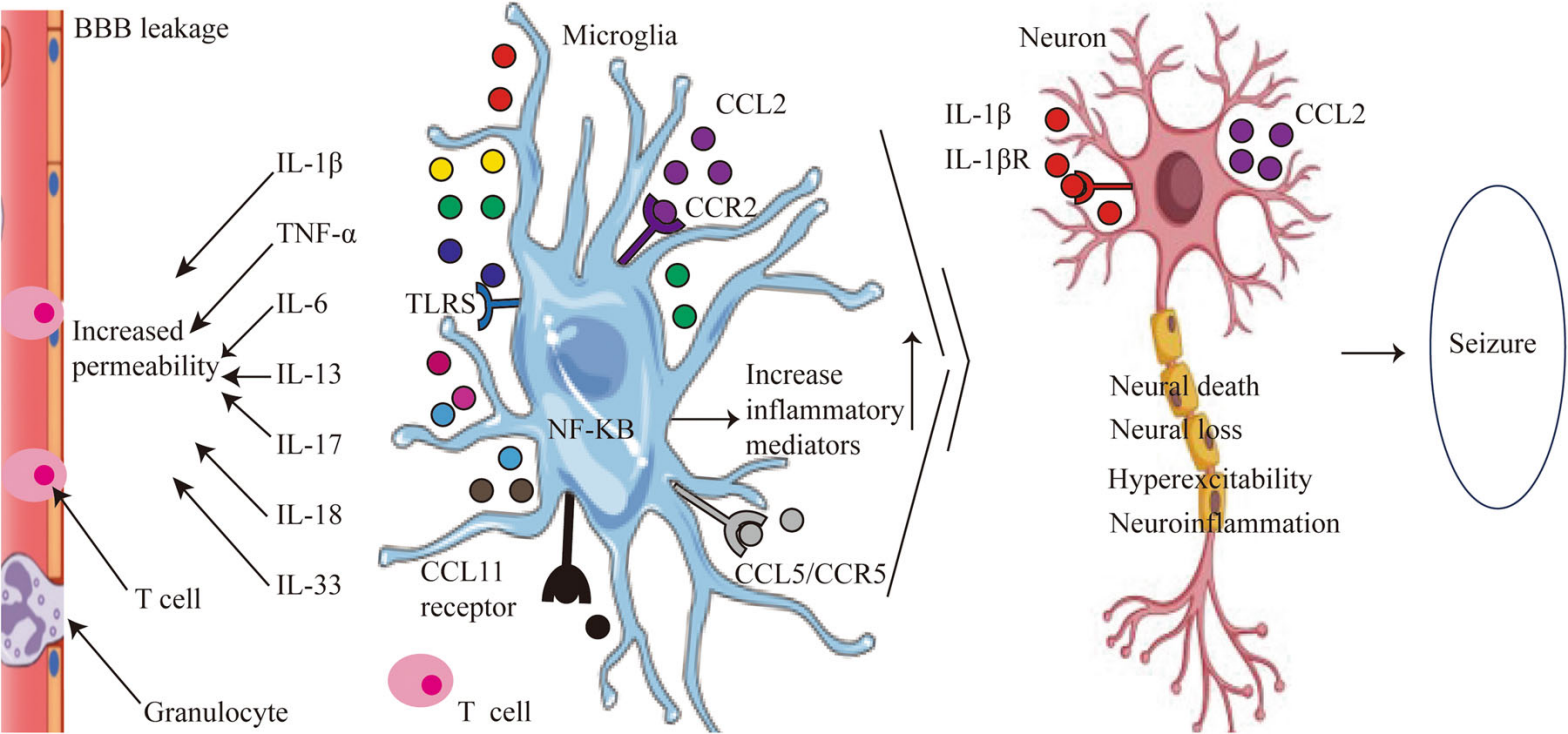
Activation of microglia in epileptic seizures. CCL promotes the activation of microglia to produce inflammatory factors, which causes damage to the blood–brain barrier, neuroinflammation, and even epilepsy. BBB leakage, the blood–brain barrier leakage; CCL, chemokine C‐C motif ligands; CCR, chemokine C‐C motif receptor; IL, interleukin; NF‐κB, nuclear factor‐kappa B; TLRS, toll‐like receptors. [Color figure can be viewed at wileyonlinelibrary.com]

Chemokines and their receptors can be produced by brain cells. Chemokine C‐C motif ligands (CCL) and chemokine C‐C motif receptors (CCR) are associated with various neurological diseases, including epilepsy.^51^ The present study has observed an increase in the expression levels of CCL2, CCL3, and CCL4 in post‐mortem hippocampus samples obtained from patients diagnosed with mesial TLE accompanied by hippocampal sclerosis.^20^ CCL2 exhibits binding affinity toward the G protein‐coupled receptor known as C‐C Chemokine receptor 2 (CCR2), which has been observed to be notably upregulated in individuals diagnosed with drug‐resistant epilepsy.^52^ The antiepileptic drugs, namely, valproate and levetiracetam can lead to a decrease in serum CCL2 levels in children with epilepsy, suggesting that CCL2 may be a potential target for epilepsy drug therapy.^53^ Crocin administration eliminates CCL4‐induced brain damage by preventing oxidative stress.^54^ But its specific mechanism is still unclear. The CCL5/CCR5 axis in many inflammatory cell types, such as microglia and astrocytes, is activated by significant peripheral inflammation, which exacerbates the breakdown of the BBB and leads to neurobehavioral dysfunction following an intracerebral hemorrhage.^55^ Notably, upregulation of CCL5 has been observed in patients with children with drug‐resistant epilepsy.^56^ Specifically, the expression of CCL5 is observed throughout the entire hippocampus during seizures. Through the blockade of CCL5/CCR5 signaling using maraviroc, microglia activation and neuron damage can be prevented in seizure mice.^57^ Activated astrocytes are responsible for the production of CCL11, while microglia primarily express the receptor for CCL11.^58^ A positive link has been observed between increased levels of CCL11 and a higher incidence of seizures,^59^ which is in line with a cross‐sectional study that reported CCL11 levels are related to clinical epilepsy severity ^60^ (Figure 1). High levels of CCL11 expression may be closely related to the occurrence of refractory epilepsy (RE) in children (Table 2).

**Table 2:** Chemokine‐related inflammatory mediator activation in clinical studies of epilepsy patients.

Chemokine	Patient population	Findings	Ref.
CCL2	Epilepsy surgery, 21 MTLE‐HS patients	Upregulation of CCL2 in MTLE‐HS patients.	[20]
CCL3	Epilepsy surgery, 21 MTLE‐HS patients	Upregulation of CCL3 in MTLE‐HS patients.	[20]
CCL4	Epilepsy surgery, 21 MTLE‐HS patients	Upregulation of CCL4 in MTLE‐HS patients.	[20]
CCL11	Patients with drug‐resistant epilepsy (*N* = 20)	Significantly increased concentrations of CCL11 and the higher level of CCL11 was correlated with an increased seizure frequency.	[59]
CXCL12	Twenty patients aged ≥50 years, ten patients were diagnosed with sAE	CXCL12 were significantly higher in the sAE group.	[33]
RANTES	25 participants underwent testing. 8 were antibody‐positive	Significant elevations in the mean concentration of RANTES in CSF were found in the antibody positive cases.	[42]

Abbreviations: CCL, chemokine C‐C motif ligand; CXCL, CXC chemokine ligand; MTLE‐HS, mesial temporal lobe epilepsy with hippocampal sclerosis; RANTES, regulated on activation, normal T‑cell expressed and secreted chemokine; sAE, suspected autoimmune epilepsy.

### 3.4 Other types of inflammatory factors

Nod‐like receptor protein 3 (NLRP3) inflammasome components are upregulated in the temporal neocortices of TLE.^61^ Moreover, a positive association has been observed between elevated levels of IL‐1β in the circulatory system and an increase in the expression of NLRP1 and NLRP3 in people diagnosed with TLE.^62^ The present study reveals that there is a significant upregulation of NLRP3 expression in the cerebral cortex of individuals diagnosed with refractory TLE, as compared to the control group. The present study investigates the potential of klotho in mitigating neuroinflammation mediated by the NLRP3 inflammasome in a rat model of TLE.^63^ The decreased level of IL‐1β expression by the inhibition of NLRP3 has the potential to ameliorate local brain damage.^25^ The binding of Signal Transduction Activator of Transcription 3 (STAT3) to the promoter region of NLRP3 enhances the acetylation of H3K9‐methylated heterochromatin, leading to increased transcription of NLRP3 and subsequent activation of NLRP3/caspase‐1‐mediated neuronal pyroptosis. This process exacerbates neuronal damage in epileptic rats.^64^

The gene Rho‐associated protein kinase 2 (ROCK2) exhibits notable upregulation in the hippocampus of patients who have received a diagnosis of drug‐resistant TLE. The expression of ROCK2 is mostly observed in astrocytes during the process of epileptogenesis. Its role in inducing epileptogenesis involves the activation of astrocyte cell cycle progression through the STAT3 pathway. The upregulated expression of ROCK2 is significantly involved in the etiology of drug‐resistant epilepsy.^65^ In this study, ROCK2 expression is first evaluated in epileptic brain tissue from patients. Hippocampal astrocytes and microglia are activated and proinflammatory cytokines are released during long noncoding RNA H19 overexpression.^66^ In a rat model of TLE, it was observed that the long noncoding RNA H19 had the ability to competitively bind to let‐7b. This binding event subsequently led to the promotion of hippocampus glial cell activation and epileptic episodes. The underlying mechanism involved the targeting of STAT3 by H19. The overexpression of let‐7b has been found to effectively hinder the activation of glial cells in the hippocampus.^67^ The findings point to a unique noncoding RNA‐mediated mechanism in seizure‐induced glial cell stimulation. On the contrary, it has been observed that miR‐21‐5p can suppress the expression of STAT3, resulting in a decrease in apoptosis, loss of hippocampus neurons, and IL‐6 levels. Consequently, this mechanism exhibits a protective influence on the hippocampal neurons of rats with epilepsy.^68^ The prevention of STAT3‐phosphorylation during the onset of epileptogenesis effectively inhibits the formation of epileptic activity patterns, cellular degeneration, the depletion of GABAergic neurons, and the sustained presence of reactive glial states.^69^ More importantly, the implementation of a targeted STAT3 knock‐out in excitatory neurons results in a decrease in the progression of seizures and hippocampal memory impairments.^70^ It is the first proof that neuronal STAT3 might affect cerebral inflammation directly. As described, blocking STAT3 may be of great significance for the treatment of epilepsy.

## 4 THE MECHANISM OF INFLAMMATORY SIGNAL PATHWAYS AND EPILEPSY

### 4.1 TGF‐β pathway

The abnormal activation of inflammatory signaling pathways is closely related to the occurrence of epilepsy. According to the literature, the TGF‐β pathway can participate in epilepsy by regulating the function of the BBB.^71^ TGF‐β pathway is mainly regulated by TGF‐β RI and TGF‐β RII. They are serine‐threonine kinase receptors, which can stimulate downstream Smad protein phosphorylation.^72^ Significantly, latent TGF‐β‐binding protein 1 (LTBP1) can affect the changes in inflammation‐related pathways by activating the TGF‐β/Smad signaling pathway and stimulate the development of epilepsy, and the regulation of epilepsy occurrence with neuroprotection can be achieved through the inhibition of LTBP1 expression.^73^ Studies have shown that inhibition of cyclooxygenase‐2 (COX‐2)‐prostaglandin E2 (PGE2) signal transduction can reduce PTZ‐induced neuroinflammation in the hippocampus, suggesting that COX‐2 promotes seizure through PGE2.^74^ However, the effectiveness of COX‐2 inhibitors is influenced by various circumstances. There is a need for future investigations to allocate greater focus toward examining the anticonvulsant properties of COX‐2 inhibitors within extensive sample sizes, employing randomized and controlled trial designs.^75^

### 4.2 NF‐κB related pathway

In a population of rats with drug‐resistant epilepsy, the expression levels of various targets in the inflammatory pathway toll‐like receptor 4 (TLR4)/nuclear factor‐kappa B (NF‐κB) are upregulated,^44^ and their drug resistance may be related to inflammatory mediators with high levels of expression. In epileptic lesions, downregulation of the inflammatory pathway TLR4/NF‐κB can inhibit the activation of microglia and the expression of inflammatory factor CD68, which can inhibit the occurrence and aggravation of epilepsy, and thus improve cognitive function and emotional disorder after seizure.^76^ Rhein has the ability to suppress the TLR4/NF‐κB signaling pathway, leading to a reduction in the secretion of inflammatory cytokines such as TNF‐α, IL‐6, IL‐1β, and IL‐18.^77^ It is noteworthy that the primary expression of interleukin‐1 receptor‐associated kinase‐M is observed in microglia, where it functions as a negative modulator of the TLR4 signaling pathway responsible for facilitating the anti‐inflammatory response.^78^ Moreover, research has demonstrated that the suppression of central IL‐1R1 effectively reduces susceptibility to seizures and ameliorates the severity of epilepsy.^79^ The deletion of the TLR3 gene can reduce the expression of TNF‐α, IL‐1, and the activity level of microglia to inhibit seizure.^80^ It is pointed out that TLR7 is widely expressed in tuberous sclerosis lesions, which is an essential cause of drug‐resistant epilepsy.^81^ TLR7 is activated in neurons in the early stage of epilepsy. TLR7 knockout significantly suppresses seizure susceptibility and neuronal excitability.^82^ However, the specific mechanism of TLR7 causing epilepsy is still unclear. The above scientific research studies suggest that downregulating the expression of TLRs and their downstream pathways may have positive implications for the treatment of epilepsy.

Correspondingly, Long noncoding RNA H19 modulates P‐glycoprotein (P‐gp) expression and neural damage in SE via the NF‐κB pathway, offering a potential medication resistance and brain damage treatment target.^83^ The expression of SerpinA3N, also known as Serpin clade A member 3 N, is notably elevated in the hippocampus of mice with TLE produced by kainic acid (KA). This increased expression is mostly observed in astrocytes. It is noteworthy to add that SerpinA3N has a substantial role in facilitating neuroinflammation induced by KA via the activation of the NF‐κB signaling pathway.^84^ HSR1101 is a promising compound to suppress migration of microglial cells and neuroinflammation, and inhibition of the mitogen‐activated protein kinases (MAPKs)/NF‐κB pathway mediates its anti‐inflammatory and anti‐migratory effects.^85^ Low‐intensity exercise combined with Sodium valproate (VPA) enhances the downregulation of NF‐κB‐related inflammatory response, thereby alleviating seizures.^86^

### 4.3 CD38/cyclic ADP‐ribose pathway

The CD38/cyclic ADP‐ribose (cADPR) pathway is also activated during epilepsy, and the CD38‐induced intracellular calcium elevation may be a critical pathological process in the development of epilepsy. The CD38/cADPR signaling pathway may be a new target for epilepsy treatment.^87^ It is reported that microglia pyruvate kinase M2 (PKM2) inhibition ameliorates neuroinflammation and neuron loss through C3‐C3aR interaction in epilepsy, which reduces the expression level of TNF‐α and IL‐1α.^88^ The results suggest that the C3‐C3aR pathway contributes to KA‐induced neurodegeneration by mediating microglia‐astrocyte communication.^89^

### 4.4 mTOR pathway

Research has found abnormal mTOR pathway activation in focal cortical dysplasia (FCD) IIB and IIA.^90^ Inhibiting the activation of the mTOR pathway in hippocampal glial cells after SE could effectively reduce neuronal damage and neuroinflammation.^91^ The enhancement of chaperone‐mediated autophagy levels by the selective inhibition of mTORC2 has the potential to ameliorate epileptic brain damage in rats,^92^ suggesting that the increased expression of the mTOR pathway in glial cells after seizure may be an important mechanism of neuronal damage. Reportedly, the upregulation of Trem2 has been shown to mitigate hippocampus neuronal damage and oxidative stress, as well as block neuronal death in epilepsy. These results are achieved through the activation of the phosphatidylinositol 3‐kinase (PI3K)/Akt pathway.^93^ Likewise, miR‐124 has been observed to exert a protective influence in the context of TLE through its facilitation of the PI3K/Akt signaling pathway, hence contributing to the preservation of cognitive function.^94^ Furthermore, the administration of glucosamine has been observed to potentially exacerbate acute and chronic epileptic convulsions in mice with epilepsy through the activation of the PI3K/Akt pathway.^95^ The expression of long noncoding RNA maternally expressed gene 3 (MEG3) has been found to have a mitigating effect on proinflammatory cytokines, oxidative stress, and apoptosis in hippocampus neurons of rats with epilepsy. The aforementioned impact is attained via triggering the PI3K/Akt/mTOR pathway.^96^ A research study discovered that the Long noncoding RNA Nespas effectively inhibits the PI3K/Akt/mTOR pathway, hence preventing the death of hippocampal neurons exhibiting epileptiform activity.^97^ Further, everolimus has the potential to diminish the PI3K/Akt/mTOR signaling pathway, mitigate neuronal death and microglia activation, and mitigate the vulnerability and intensity of seizures.^98^ More research with a larger sample size is needed to provide more information and statistical credibility.

### 4.5 Janus kinase‐signal transducer and transcriptional activator (JAK‐STAT) signaling pathway

Meanwhile, the JAK‐STAT signaling pathway has been found to exhibit a significant correlation with numerous immunological and inflammatory illnesses. The suppression of α‐synuclein‐induced microglia and macrophage activation, as well as the migration of CD4^+^ T‐cells into the central nervous system, ultimately leads to the inhibition of neurodegeneration. This effect is achieved through the inhibition of the JAK/STAT pathway, which has an impact on both innate and adaptive immune responses.^99^ Genistein exhibits the capacity to hinder the JAK2‐STAT3 inflammatory pathway and inhibits the expression of apoptotic proteins, hence resulting in an increase in the number of viable neurons.^100^ The prevention of STAT3‐phosphorylation blocking in the acute phase of epileptogenesis inhibits the formation of epileptic activity patterns and the occurrence of overall cell loss.^69^ Further, the implementation of a targeted STAT3 knock‐out specifically in excitatory neurons leads to a decrease in the course of seizures and the manifestation of memory impairments in the hippocampus.^70^

### 4.6 Nrf2‐related pathway

Recent research shows that following the onset of SE, the activation of nuclear factor erythroid‐derived 2‐related factor 2 (Nrf2) primarily occurs in the hippocampus and persists throughout the whole duration of epileptogenesis.^101^ The expression of Nrf2 and the associated downstream genes exhibits a transitory rise, reaching its peak during the early stages following the seizure, primarily in the hippocampus.^102^ Carveol functions as a stimulator of Nrf2, hence initiating the production of antioxidants and alleviating inflammatory damage through many pathways.^103^ Sulforaphane has been found to augment the expression of Nrf2 and associated antioxidant genes, hence increasing the overall antioxidant capacity in both the plasma and hippocampus. After experiencing a traumatic brain injury, the neuroprotective impact of Nrf2 activation has been observed through the reduction of neuronal cell death and enhancement of antioxidant capacity.^104^ Ubiquitin‐specific peptidase 15 (USP15) inhibition induces Nrf2 nuclear translocation and promotes heme oxygenase protein expression level. The potential therapeutic benefit of pharmacologically inhibiting USP15 in the context of alleviating epileptic seizures may be attributed to its ability to counteract oxidative damage.^105^ Through the Nrf2‐mediated NLRP3 and NF‐κB pathways, hydrogen reduces cell damage, apoptosis, inflammation, and oxidative stress.^106^ Correspondingly, salidroside treatment has the potential to induce the upregulation of nuclear factor erythroid 2‐related factor‐antioxidant response element (Nrf2/ARE) signaling pathways which have been discovered to be involved in the suppression of oxidative stress response and neuroinflammation.^107^ The neuroprotective effects of ginsenoside Rb1 have been seen in the context of brain damage generated by PTZ and neuron injury induced by Mg2+ free. It is believed that these effects are mediated by the activation of the Nrf2/ARE signaling pathway.^108^ IL‐1‐Exo inhibited lipopolysaccharide‐induced inflammatory responses in astrocytes and animals with SE. Furthermore, it has been determined that the primary mechanism by which IL‐1‐Exo exerts its effects is through the activation of the Nrf‐2 signaling pathway.^109^ The expression of the Nrf2/ARE signaling pathway has been shown to mitigate the pathological damage shown in rat hippocampus neurons. Additionally, it has been found to extend the latency period of seizures and decrease the severity of epileptic seizures in rats.^110^

### 4.7 Wnt3a/β‐catenin signaling pathway

According to the literature, Wnt3a/β‐catenin signaling functions as a connection between abnormal neurogenesis and the underlying remodeling processes occurring in the hippocampus, ultimately resulting in the development of TLE.^111^ Wnt/β‐catenin signaling is downregulated in the acute stage of status epilepsy.^112^ Chronic intermittent hypobaric hypoxia (CIHH) has been found to effectively alleviate impairments in spatial and object memory, hippocampus neurogenesis, and synaptic plasticity in rats with epilepsy treated by pilocarpine. The restoration of cognitive deficits in epileptic rats is achieved through the stimulation of the Wnt/β‐catenin pathway by CIHH.^113^ The molecular mechanisms of how CIHH regulates the Wnt/β‐catenin pathway remain unclear. The prevention of aberrant proliferation of neural progenitors in the epileptic hippocampus is achieved by the inhibition of the Wnt/β‐catenin pathway.^114^ Overall, the process of hippocampal neurogenesis during epilepsy was mediated by the Wnt/β‐catenin signaling system, which may offer novel approaches to the management of TLE.

### 4.8 Programmed cell death 1 (PD‐1)‐related pathway

Compared to patients experiencing partial seizures, those with intractable SE showed a more marked increase in CSF‐ and serum‐PD‐1 levels. The measurement of serum and CSF levels of PD‐1 holds promise as a potential clinical diagnostic biomarker for intractable epilepsy.^115^ PD‐1 levels are upregulated in the specimens of surgically resected specimens from patients with intractable epilepsy, and anti‐PD‐1 treatment protects against seizures by suppressing sodium channel function.^116^ Samples from the brains of nine people who had RE and undergone neurosurgery were observed. Further clinical research with a larger sample size is needed to provide more information and statistical credibility.

## 5 TREATMENTS OF REFRACTORY EPILEPSY BASED ON ANTI‐INFLAMMATORY STRATEGY

Although many patients with epilepsy will achieve seizures controlled with antiseizure medications, a large percentage of patients are refractory to drug therapy for polydrug toxicity and psychiatric and cognitive comorbidities. Even after going through three different generations of antiepileptic drugs, some people still suffer from drug‐resistant epilepsy. These patients are usually prescribed a higher dose of antiepileptic drugs, which results in more adverse effects. The ketogenic diet (KD) has been the subject of extensive research for over a century, particularly in relation to its impact on neurological disorders such as epilepsy. The utilization of a KD for the management of epilepsy is associated with several key factors, including the mitigation of reactive oxygen species production, the reduction of neuronal inflammation, and the restoration of neuronal myelin sheath.^117^ In childhood drug‐resistant epilepsy, KD has been shown to be a viable and secure nonpharmacological and nonsurgical treatment option for the management of childhood drug‐resistant epilepsy. It has demonstrated good effects on growth and electroencephalographic activity.^118^ Children with RE benefit greatly from KD treatment, which is effective and produces a high retention rate. In RE children, the short‐term effectiveness of KD is influenced by magnetic resonance imaging (MRI) abnormalities, beginning age, and duration.^119^ Although the frequency of seizures may remain the same or decrease while on the KD, around two‐thirds of patients are able to reduce their antiseizure medication after starting the KD.^120^ The KD algorithm offers a methodical framework for administering the KD and has exhibited favorable health effects in pediatric patients.^121^ It is reported that KD therapy has been found to be a safe and efficacious therapeutic option for both Chinese adults and children who are diagnosed with drug‐resistant epilepsy.^122^ Clinical trials with long‐term follow‐up are needed to evaluate the efficacy of KD. Correspondingly, in contrast to the control group, the modified Atkins diet (MAD) group exhibits notable enhancements across all dimensions, encompassing seizure frequency and behavioral difficulties.^123^ In the short term, all dietary therapies are successful. In contrast, MAD has superior tolerability and a higher seizure decrease, which makes MAD a more viable alternative than KD.^124^ The utilization of the MAD has demonstrated efficacy in the management of seizures. However, further study is necessary to evaluate the effectiveness of the intervention in relation to biomarkers, as well as to conduct descriptive metabolomics studies.^125^

In physical therapy, vagus nerve stimulation can downregulate the expression of inflammatory mediator. The antiepileptic mechanism of vagus nerve stimulation may be achieved by inhibiting the expression of inflammatory mediators in epileptic foci.^47^ In drug‐resistant epilepsy, the stimulation of the vagus nerve has a notable impact on the activity of brain networks, as evaluated using electroencephalography. This stimulation affects a broad distribution of networks throughout the brain.^126^ A clinical study suggests that the frequency of seizures is notably reduced after intermittent stimulation of the vagus nerve.^127^ In drug‐resistant epilepsy patients, instantaneous vagus nerve stimulation also reduces seizures and improves cognition.^128^ Although vagus nerve stimulation therapy is currently being applied to pediatric patients with a high degree of safety and efficacy,^129^ for patients with drug‐resistant epilepsy, further clinical research with a larger number of samples is needed. Besides, its potential mechanism is waiting for exploration.

**Figure 2:**
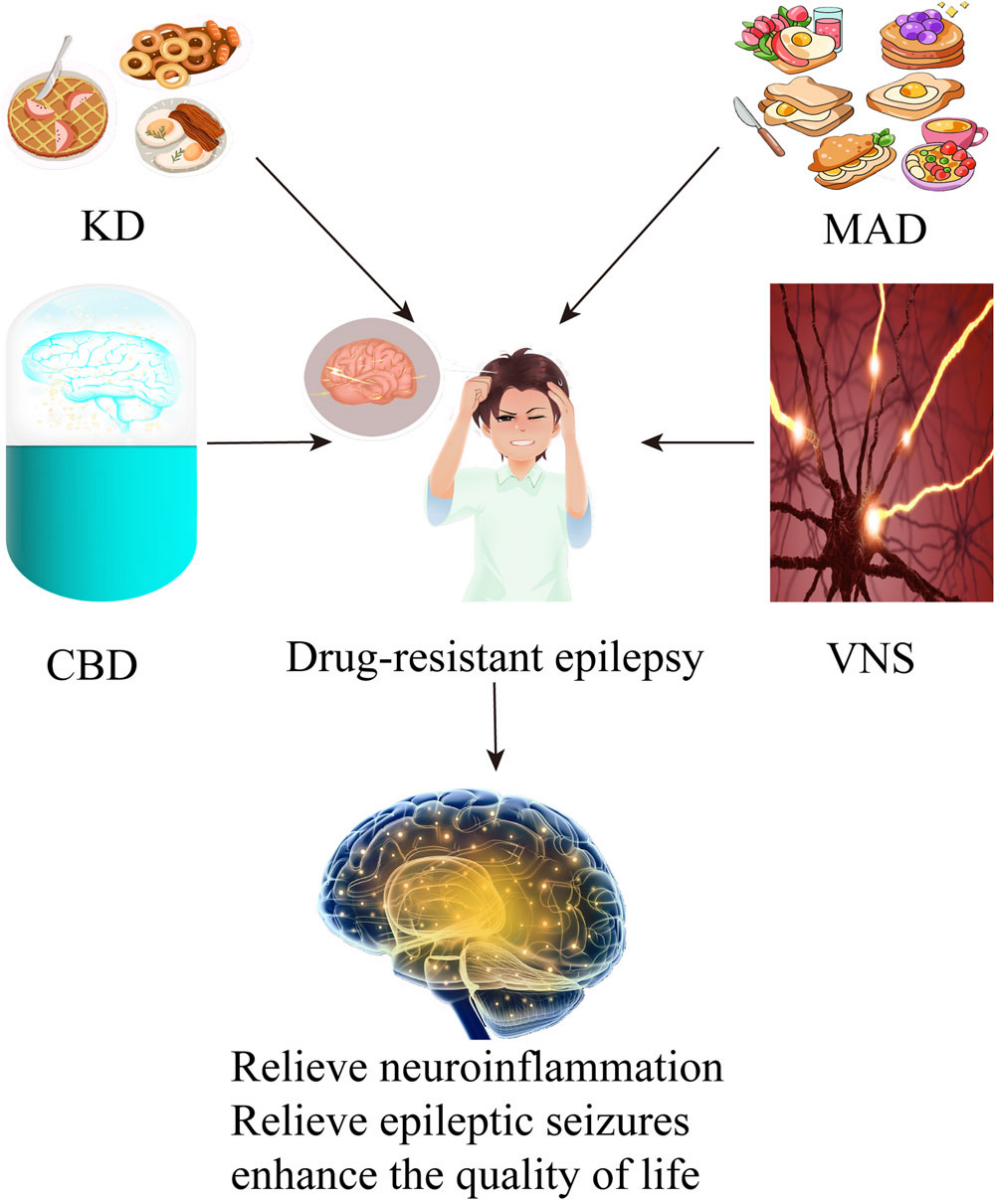
Treatment of refractory epilepsy based on anti‐inflammatory strategy. CBD, Cannabidiol; KD, ketogenic diet; MAD, the modified Atkins diet; VNS, vagus nerve stimulation. [Color figure can be viewed at wileyonlinelibrary.com]

In drug therapy, Cannabidiol (CBD) exhibits potent anti‐inflammatory and neuroprotective properties, which potentially play a role in the protective benefits observed in epilepsy and other related disorders. A few clinical trials have substantiated the efficacy of CBD as a treatment for epilepsy.^130^ The utilization of cannabis‐based magistral formulation has demonstrated significant efficacy and safety in the treatment of drug‐resistant focal epilepsy among adult patients. The sustained decrease in the incidence of seizures is observed over an extended time.^131^ The efficacy, safety, and high degree of tolerance of CBD as an additional therapy in adult patients diagnosed with drug‐resistant focal epilepsy have been proven. Moreover, this therapeutic approach has been found to be significantly correlated with an enhancement in the patient's overall quality of life.^132^ CBD has exhibited a high level of safety and efficacy as an antiseizure medication, displaying a wide range of effectiveness in treating various epileptic types, including those linked to severe forms of childhood‐onset epilepsies^133^ (Figure 2). It is reported that liraglutide can also reduce SE‐induced chronic inflammation and mitochondrial damage, suggesting that liraglutide can heal and protect the brain after SE, making it a viable treatment.^134^ Further, animal experiments have shown that liraglutide reduces spike percentages in PTZ‐induced epilepsy. Additionally, liraglutide significantly lowers TNF‐α and IL‐1β levels.^135^ However, there is no clinical study about the therapeutic effect of liraglutide on epilepsy.

## 6 CONCLUSION

In summary, the involvement of inflammatory mediators and their associated pathways is crucial in the etiology and progression of epilepsy. The benefits of anti‐inflammatory medication in drug‐resistant epilepsy suggest that targeting the inflammatory response inside the central nervous system holds promise as a novel avenue for forthcoming epileptic treatments. Although significant progress has been made in the research on the mechanisms of inflammation and inflammatory molecules, the mechanism of drug‐resistant epileptogenesis has not been fully elucidated yet. Additional study is required to investigate the involvement of inflammatory mediators and their pathways in the etiology and progression of epilepsy and establish novel theoretical frameworks and therapeutic strategies for the prevention and management of epilepsy.

## AUTHOR CONTRIBUTIONS

Yue Yu contributed to collecting literature and writing the original draft. Fei‐Ji Sun contributed to writing and revising the manuscript.

## CONFLICT OF INTEREST STATEMENT

The authors declare no conflict of interest.

## ETHICS STATEMENT

Not applicable.

## Data Availability

Not applicable as no new data are generated in this study.
